# The Treatment of Lobar Pneumonia in Children

**Published:** 1905-11-18

**Authors:** 


### THE TREATMENT OF LOBAR PNEUMONIA IN CHILDREN.

In two interesting lectures on lobar pneumonia in children Dr. Geo. A. Sutherland [1] discusses the question of treatment, and his conclusions may here be briefly summarised. Granted favourable conditions, such as rest in bed, fresh air and suitable diet, the disease in most instances will run its course to a favourable termination. The establishment of these conditions, the provision of good nursing, and the prescription of some simple febrifuge mixture, is in many cases an adequate scheme of treatment, though careful observation of the patient is always necessary even in cases of favourable appearance. Care is needed (1) to relieve troublesome and weakening symptoms, and (2) to treat any complications which may arise. There are numerous individual symptoms which require more or less special care and treatment. For example, pleuritic pain may cause the child much suffering. To meet this such a local application as a turpentine fomentation or a linseed-meal poultice may be applied for half an hour or so. In the severe pain due to diaphragmatic pleurisy two or three leeches at the site of the pain and strapping of the affected side are often very successful. These failing, opium or morphine must be prescribed. As a general rule opium is contraindicated in pneumonia because of its depressing action on the respiratory centre. But in very severe pain the suffering and restlessness are dangers in themselves and may well be more prejudicial to the patient than one or two doses of morphine. Another symptom which causes a good deal of disturbance is cough. This is usually severe in direct proportion to the amount of lung involved and may be short and hacking in pure pneumonia or more or less bronchial from the presence of catarrh. Local hot applications as above described are here again useful as soothing agents. The position of the patient in bed should be studied, as it is often found that some change of position is followed by cessation of the cough. Steam inhalations used for ten to fifteen minutes and at intervals of several hours are often beneficial. Continuous steaming, like continuous poulticing, is not practised in modern treatment. Belladonna, five to ten minim doses of the tincture, is a useful medicine for checking cough, as is also paregoric. In dealing with the pyrexia that accompanies pneumonia, it must be remembered that 103° to 104.5° is an ordinary evening record in children and that it demands no treatment; even 105° or 106° may be noted without the presence of any sign of distress, and in such circumstances there is no claim for interference. But when with high temperatures there are such symptoms as restlessness and sleeplessness, special measures are called for. In infants a hot bath which is rapidly cooled to 85°, is often successful both in getting rid of the restlessness and reducing the temperature. In older children cold or tepid sponging is a suitable measure, and if there are marked nervous disturbances cold should be applied to the head. A hot fomentation and one drachm doses of solution of ammonium acetate is a useful antipyretic combination. A point to remember is that hyperpyrexia may be due to some event quite independent of the pneumonia, such as gastro-intestinal disturbance or acute otitis media. Another symptom which may require special treatment is sleeplessness. This is often accompanied by active delirium and leads to marked prostration. Here again the possibility of acute middle ear inflammation should be borne in mind. Bromide of ammonium in ten grain doses, bromidia (fifteen minims), also veronal and sulphonal are useful remedies, but opium should not be used. Brandy, two to four drachms, in hot water often acts like a charm. Other measures are the application of cold water to the head or hot water to the chest, but too much fussing about the patient may keep up the excitement and so prevent sleep. Lastly may be mentioned the aggravation of the patient's condition which is seen shortly before the occurrence of the crisis and which often needs special therapeutic measures. At this time signs of cardiac weakness must be carefully watched for and an endeavour must be made to determine whether the failure is primarily in the right or the left heart. Evidence of dilatation of the left ventricle are extension outwards of the apex impulse, weakness of the first sound at the apex and a tendency to syncope. These call for brandy, strychnine and ether in doses increasing until improvement is manifested. In urgent cases strychnine should be given hypodermically and in relatively large doses, as in the toxæmia of pneumonia the tissues are much less sensitive to the drug than at other times. To an infant a year old a minim of the "liquor" may be given by the mouth or half a minim hypodermically; these amounts may be repeated every four hours. Signs of failure of the right ventricle are cyanosis, dyspnœa, and over action of the right heart; this condition is also signalled by gradual enlargement of the liver, and often also by the physical signs of œdema of the lung. The claim here is for bleeding by three or four leeches applied in the hepatic region and followed by a hot fomentation. Calomel and saline purgatives will also help to relieve the venous fulness, and alcohol and strychnine may be ordered as cardiac tonics. Dry cupping over the bases of the lungs is specially useful when pulmonary engorgement is present. When cyanosis is observed the inhalation of oxygen or of a superoxygenated atmosphere may be of service. For respiratory distress not obviously dependent on the state of the lungs or heart and probably due to failure of the respiratory centre from toxæmia, atropine may be given with strychnine as a direct respiratory stimulant. A dose of $\frac{1}{100}$ grain may be ordered every four hours to a child of five years.

[1] Clin. Jour., Nov. 1, 1905.

